# Cytomegalovirus Antibody Elevation in Bipolar Disorder: Relation to Elevated Mood States

**DOI:** 10.1155/2015/939780

**Published:** 2015-05-13

**Authors:** A. R. Prossin, R. H. Yolken, M. Kamali, M. M. Heitzeg, J. B. Kaplow, W. H. Coryell, M. G. McInnis

**Affiliations:** ^1^Department of Psychiatry, University of Texas Health Science Center at Houston, 1941 East Road, No. 2308, Houston, TX 77054, USA; ^2^Department of Symptom Research, University of Texas MD Anderson Cancer Center, Houston, TX 77030, USA; ^3^Stanley Laboratory of Developmental Neurovirology, Johns Hopkins University Medical Center, Baltimore, MD 21287, USA; ^4^Department of Psychiatry, University of Michigan Medical School, Ann Arbor, MI 48109, USA; ^5^Department of Psychiatry, University of Iowa School of Medicine, Iowa City, IA 52242, USA

## Abstract

The neurobiology of mood states is complicated by exposure to everyday stressors (e.g., psychosocial, ubiquitous environmental infections like CMV), each fluctuating between latency and reactivation. CMV reactivation induces proinflammatory cytokines (e.g., TNF-*α*) associated with induction of neurotoxic metabolites and the presence of mood states in bipolar disorder (BD). Whether CMV reactivation is associated with bipolar diagnoses (trait) or specific mood states is unclear. We investigated 139 BD type I and 99 healthy controls to determine if concentrations of IgG antibodies to Herpesviridae (e.g., CMV, HSV-1, and HSV-2) were associated with BD-I diagnosis and specific mood states. We found higher CMV antibody concentration in BD-I than in healthy controls (T_234_ = 3.1, *P*_uncorr_ = 0.002; *P*_corr_ = 0.006) but no difference in HSV-1 (*P* > 0.10) or HSV-2 (*P* > 0.10). Compared to euthymic BD-I volunteers, CMV IgG was higher in BD-I volunteers with elevated moods (*P* < 0.03) but not different in depressed moods (*P* > 0.10). While relationships presented between BD-I diagnosis, mood states, and CMV antibodies are encouraging, they are limited by the study's cross sectional nature. Nevertheless, further testing is warranted to replicate findings and determine whether reactivation of CMV infection exacerbates elevated mood states in BD-I.

## 1. Introduction

Bipolar disorder (BD) a mood disorder characterized by the presence of elevated, irritable, or mixed mood episodes frequently interspersed with episodes of depression affects approximately 2-3% of the population [1–3]. Despite substantial individual and societal impact, knowledge of the biological processes underlying and driving mood states in BD is limited. Revealing associations between biological factors and both mood traits and states will set a trajectory for understanding the pathophysiology of moods and in developing novel, more efficacious intervention strategies in BD.

Ubiquitous environmental infections (e.g., Herpesviridae including cytomegalovirus; CMV) and associated human immune responses fluctuate between latency and reactivation in humans, potentially triggered by psychosocial stressors [4–6]. Viruses may facilitate exacerbation of psychiatric disease pathology through various mechanisms, including induction of inflammatory factors (e.g., TNF-*α*, IL-6, etc.) [7] or via direct interactions with specific illness susceptibility genes. A recent preliminary fMRI study of pediatric bipolar disorder may suggest a mechanism whereby alterations in TNF-*α* related processes could impact some of the symptoms in BD-I. In this study, Barzman et al. identified correlations between 11 TNF-*α* related gene expressions and activation within the amygdala or anterior cingulate cortex during the affective Posner task [8]. Evidence from recent studies in BD also shows that TNF-*α* is higher in BD volunteers compared to healthy control volunteers [9, 10]. Further, as outlined in a review by Brietzke et al., existing evidence suggests that TNF-*α* is higher in the midst mood episodes in BD volunteers as compared to healthy control volunteers [10, 11].

Amongst the potential viral candidates, the herpesvirus family has received the most attention. Certain Herpesviridae (e.g., HSV-1) have been associated with clinical features of BD [12], but to date, no studies have determined whether CMV is associated with the presence of either mania or depression in BD. However, evidence from volunteers with schizophrenia, a psychiatric illness sharing certain clinical and biological features with BD (see review by Prossin and colleagues) [13], suggests that* Cytomegalovirus* (CMV) may interact with certain high risk genetic loci to precipitate schizophrenia illness [14–18].

Following the diathesis-stress model of disease [19], exposure to environmental stressors (psychosocial, behavioral, and biological) could potentially increase risk for psychiatric illness, particularly in individuals at high genetic risk for that illness [20, 21]. However, while knowledge of behavioral phenotype in BD has grown [22], facilitating development of more efficacious behavioral interventions (e.g., interpersonal and social rhythm therapy) [23], the biologically based environmental factors contributing to BD remain elusive. Discovery of such factors will facilitate development of novel, personalized, immune-based treatment strategies in this debilitating, life-threatening illness. Here, in cross sectional analyses of volunteers enrolled in a longitudinal study of bipolar disorder, we test our hypotheses that BD volunteers have higher Herpesviridae (e.g., CMV, HSV-1, and HSV-2) IgG concentrations compared to healthy control volunteers and that concentration of these antibodies is associated with common behavioral phenotypes in BD, elevated and/or depressed mood state(s).

## 2. Materials and Methods

The study was approved by the University of Michigan Investigational Review Board. Written informed consent was obtained from all study participants.

We randomly selected 238 volunteers between 18 and 65 years of age from the Prechter Bipolar Longitudinal Study (139 with BD-I and 99 healthy controls). Volunteers either met DSM IV [24] criteria for BD-I (at least one prior primary manic and/or mixed episode) (with or without comorbid substance use, other psychiatric disorders) or were healthy controls, without mental health diagnoses (on either axis I or axis II) [24]. DSM IV [24] diagnoses were assessed using the Diagnostic Inventory for Genetic Studies (DIGS) [25]. Following the diagnostic interview, all volunteers, provided they do not withdraw consent, remain in the Prechter Bipolar Longitudinal Study regardless of the diagnosis(es) determined. For the current study, we selected 238 volunteers who were actively participating in the Prechter Longitudinal Study. Prechter Bipolar Longitudinal Study volunteers routinely return to the research center for follow-up assessments, including longitudinal diagnostic confirmation and mood assessments. Upon their return to the center, subjects were chosen for the current study based on their availability/consent for blood sampling on a first come first serve basis and within a limited time frame. All volunteers for the current study, including both healthy control volunteers and BD-I volunteers, were actively participating in the Prechter Bipolar Longitudinal Study.

Additional mood measures including Hamilton Depression Rating Scale (HDRS) [26] and Young Mania Rating Scale (YMRS) [27] were completed during assessments, consistent with time of blood sampling. The YMRS and HDRS were dichotomized into clinically elevated mood (YMRS > 7), clinically depressed mood (HDRS > 7), and clinically mixed mood (YMRS > 7 and HDRS > 7).

Overall, the mean age of volunteers was 36 ± 14 years of age. Study entry was not constrained by either body mass index (BMI) or sex. Based on evidence implicating their potential impact on immune functioning these variables were entered into analyses to test for individual effects of age, sex, or BMI on CMV IgG. Diagnostic breakdown of anthropometric and sociodemographic variables is included in Table 1. Whether or not patients were treated with psychotropic medications (i.e., lithium, lamotrigine, valproate, carbamazepine, atypical antipsychotic, or antidepressant medication) was included in analyses as a dichotomous variable, medication usage.

Whole venous blood was sampled following completion of psychiatric assessments at 1 PM (±1 hour). Samples were centrifuged for 15 minutes at 4750 rpm and plasma was extracted and stored at −80°C. Serology assessments were performed at the Stanley Neurovirology Laboratory (Johns Hopkins University School of Medicine, Baltimore, MD). CMV IgG antibody concentrations were expressed quantitatively as both continuous (e.g., concentration) and dichotomous (e.g., seropositive, seronegative) measures, each derived via comparisons to standard samples run concurrently in each assay, as previously described [28]. Similar processes were completed for quantification of Herpes Simplex Virus Type I (HSV-1) and Herpes Simplex Virus Type 2 (HSV-2).

## 3. Data Analytic Plan

SPSS Statistics software version 21 (IBM Inc., Chicago, IL) was used to plot the data, rule out the presence of outliers, and perform additional statistical analyses. Medication usage (described above) was used to rule out overt effects of psychotropic medications on CMV IgG. Planned analyses included usage of independent samples T-tests to detect diagnostic differences in viral antibody concentrations (CMV, HSV-1, and HSV-2). Subsequent analyses used separate independent samples T-tests to identify whether CMV antibody concentrations were higher in BD-I volunteers in the midst of a mood episode (i.e., with or without a depressed mood episode, with or without a manic mood episode) while controlling for covariates (age, sex, race, and BMI). Subsequently, we used the Pearson chi-squared test, to show that CMV antibody status was similar in BD-I groups with and without current psychotropic treatment. Separate Spearman correlation analyses (and independent samples T-testing) tested for the presence of linear relationships between CMV antibody concentrations and either age, BMI, sex, or medication treatment, all factors potentially associated with variation in immune activation, to determine whether these factors were likely confounding our results. Data shown are corrected for multiple comparisons where indicated by “*P*_corr_” using the Bonferroni technique [29] (uncorrected *P*-values are simply stated as “*P* = ”). Statistical significance for all analyses was set at *P* = 0.05.

## 4. Results

In total, plasma samples from 238 study volunteers were assayed for CMV, HSV-1, and HSV-2 antibodies. Of these 238 volunteers, 139 had a diagnosis of BD-I and 99 were healthy controls. Of the 139 BD-I volunteers, sixty-seven BD-I individuals (48%) exhibited evidence of clinically significant mood symptoms (e.g., depressed, elevated, and mixed). Sixty-one BD-I individuals (44%) had clinically significant depression, 22 BD-I individuals (16%) had clinically significant mood elevation, and 13 BD-I individuals (9%) had symptoms consistent with that of a mixed mood state. Mean values of behavioral measures of interest (e.g., YMRS, HDRS) are presented for each diagnostic study group (e.g., BD-I, healthy controls) in Table 2.

**Figure 1:**
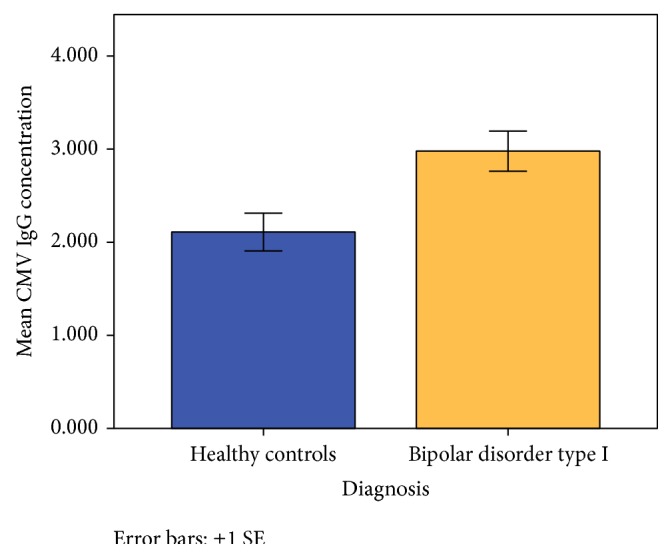
Graphical depiction of diagnostic differences in* Cytomegalovirus* (CMV) antibody concentrations. Standardized mean antibody (IgG) concentrations are depicted on the vertical, *y*-axis and diagnosis on the horizontal *x*-axis. CMV IgG concentration was higher in BD-I volunteers (shown in maize color) as compared to healthy control volunteers (shown in blue color) (T_234_ = 3.1; *P*_uncorr_ = 0.002; *P*_corr_ = 0.006). No diagnostic differences were identified with regard to HSV-1 IgG (T_236_ = 0.15; *P* = 0.89) and HSV-2 IgG (T_236_ = 0.14; *P* = 0.89). Error bars represent ±1 standard error.

Using independent samples T-testing, we found that volunteers with a diagnosis of BD-I have significantly greater CMV IgG concentrations (T_234_ = 3.1; *P* = 0.002; *P*_corr_ = 0.006; mean difference 1.0 ± 0.3) as compared to healthy control volunteers. However, no diagnostic differences were identified with regard to HSV-1 IgG (T_236_ = 0.15; *P* = 0.89) and HSV-2 IgG (T_236_ = 0.14; *P* = 0.89). Graphical depiction of diagnostic differences in viral antibody concentrations is illustrated in Figure 1; statistical comparisons involving CMV IgG are outlined in Table 3.

Separate independent samples T-tests found that CMV IgG concentrations were higher in those BD-I volunteers in the midst of a clinically elevated mood state (T_132_ = 2.2; *P* = 0.03; mean difference 1.3 ± 0.6) but not significantly different in those BD-I volunteers in a clinically depressed mood state (*P* > 0.10) as compared to those study volunteers not in a clinically elevated mood state or clinically depressed mood state, respectively.

Results from individual Spearman correlational testing showed that CMV antibody concentrations were not significantly correlated with either body mass index (*P* > 0.10) or age (*P* > 0.10) in BD-I volunteers. Additionally in BD-I volunteers, using separate independent samples T-tests, we found that (1) CMV antibody concentrations were not significantly different in females as compared to males (*P* > 0.10) and (2) CMV antibody concentrations were not significantly different in volunteers who were being treated with psychotropic medications as compared to volunteers not receiving psychotropic medication treatment (*P* > 0.10).

## 5. Discussion

This study identified an association between concentrations of plasma CMV IgG antibodies and a diagnosis of BD-I, with BD-I individuals having significantly higher CMV concentrations than healthy control volunteers. Further, chi-squared testing described in Table 3 showed that CMV IgG seropositivity was associated with greater than 5 times increased likelihood of having a diagnosis of BD-I (Table 3). This finding aligns with previous findings of CMV IgG seropositivity in psychiatric disorders, specifically schizophrenia [14–18], and supports the hypothesis that exposure to environmental/infectious factors like viruses may contribute to the pathophysiology of BD-I. While recent evidence showed that passage of maternal CMV antibodies to the neonate in expecting mothers was not shown to pose significant risk of BD-I in the neonate [30], results we present of associations between CMV antibody concentrations, diagnosis of BD-I, and elevated mood state warrant further investigation and clarification on questions of causality. Approximately 50% of Americans are seropositive for CMV [31]. Initial exposure to infectious agents like CMV induces an immune response [32], memory of the infection persisting in the form of CMV IgG antibodies. Subsequently, CMV persists in a latent state in immature cells [33]. Exposure to psychosocial stress can potentially downregulate cellular immune responsivity [34–36] reactivating otherwise latent herpesviruses (e.g., CMV) [37], inducing herpesvirus (e.g., CMV) antibodies [5]. Taken together with the findings we report this evidence suggests that treatment of ubiquitous, asymptomatic herpesvirus infections or targeting their downstream counterparts (e.g., TNF-*α*) could potentially impact the BD-I illness. However, much further testing on expanded, longitudinal BD-I samples is required to test these hypotheses. Separate evidence does show that elevation of soluble CMV antigens and CMV antibody concentrations is associated with a shift towards CD8^+^ T-cell production [32] and subsequent induction of CD8^+^ derived proinflammatory cytokines (e.g., TNF-*α*, IL-6, and IFN-*γ*) [7, 38, 39]. Further, enhanced immune activation involving elevated concentrations of these cytokines has been identified in BD volunteers [40] and phasic variation in common clinical features of BD (e.g., depression, mania, suicidality, etc.) has also been associated with particular inflammatory cytokine profiles [41]. Induction of TNF-*α* has been shown to modulate neurotransmitter metabolism via activation of indoleamine 2,3-dioxygenase (IDO), subsequently reducing neurotransmitter precursors (i.e., tryptophan), and shifting the balance towards production of potentially neurotoxic metabolites (i.e., hydroxykynurenine) [42–45]. The resulting alteration of neurotransmitter metabolism is believed to directly impact central processing of emotionally salient and stressful events, resulting in altered behavioral response to stress [46–51].

In summary, these results augment accumulating evidence suggesting that exposure to Herpesviridae in general (and CMV in particular), its subsequent acquired immune response, and the impact of psychological stressors on immune reactivation may pose a risk of BD-I, potentially via an impact on development or exacerbation of elevated mood states. However, the cross sectional nature of analyses involving CMV in our study limits the extent that causal inferences can be drawn. Potentially, the means by which episodic inflammatory alterations contribute to episodic clinical features in BD could be ascribed to this model but further research in this area is required to discern the exact mechanisms underlying CMV's relationship to clinical mood states in BD-I. Additionally, while we found no significant differences in CMV antibody concentrations when comparing BD-1 volunteers receiving pharmacotherapy against those not receiving pharmacotherapy, it remains as a possibility that interindividual variation in specific psychiatric medication used (and/or specific dosage prescribed) could be confounding the results. Future studies that include designs with an expanded population of BD-I volunteers will be better positioned to test for the presence of medication specific effects on CMV antibody concentrations.

## Figures and Tables

**Table 1:** Here we provide demographic and anthropometric information on study volunteers.

Diagnosis	Age	Sex		Race						Body mass index (BMI)
	Mean ± SD (*n* = 237)	Male (*n* = 101)	Female (*n* = 137)	American Alaskan Native	Asian	Black African American	White Caucasian	More than one race	Unknown	Mean ± SD (*n* = 130)
Bipolar disorder type I	39 ± 13 (*n* = 138)	37% (*n* = 52)	63% (*n* = 87)	0.7% (*n* = 1)	2.9% (*n* = 4)	3.6% (*n* = 5)	87.1% (*n* = 121)	4.3% (*n* = 6)	1.4% (*n* = 2)	29 ± 8 (*n* = 132)

Healthy control	32 ± 14 (*n* = 99)	49% (*n* = 49)	51% (*n* = 50)	0% (*n* = 0)	12.1% (*n* = 12)	15.2% (*n* = 15)	68.7% (*n* = 68)	4.0% (*n* = 4)	0% (*n* = 0)	26 ± 6 (*n* = 98)

Results are presented as mean ± standard deviation for body mass index (BMI) and as percentages for other variables within each diagnostic group. These measures include age, sex, race, and body mass index.

**Table 2:** Here we provide clinical information on study volunteers.

Diagnosis	HDRS	YMRS	Medication use					
	Mean ± SD (*n* = 235)	Mean ± SD (*n* = 230)	AAP's	Antidepressant	Lithium	Valproate	Lamotrigine	Carbamazepine
Bipolar disorder type I	8.1 ± 7.6 (*n* = 137)	3.6 ± 5.6 (*n* = 134)	45%	50%	36%	20%	24%	5%

Healthy control	0.7 ± 1.3 (*n* = 98)	0.1 ± 0.3 (*n* = 96)	0%	0%	0%	0%	0%	0%

Results are presented as mean ± standard deviation (and percentage of volunteers using a particular medication) within each diagnostic group. These measures include Hamilton 17-item Depression Rating Scale (HDRS), Young Mania Rating Scale (YMRS), and medication use including AAP's (atypical antipsychotics), antidepressants, lithium, valproate, lamotrigine, and carbamazepine.

**Table 3:** Here we provide CMV IgG concentrations (reported as mean ± standard deviation in column 2) for each diagnosis, BD-I and healthy controls.

Diagnosis	CMV IgG concentrations	Diagnostic comparison of CMV	CMV IgG seropositivity		Pearson chi-square
	Mean ± SD	Antibody concentrations	Seronegative	Seropositive	Testing
Healthy control volunteers	2.1 ± 2.1 (*n* = 99)	T_234_ = 3.1 *P* = 0.002	*n* = 59	*n* = 40	Likelihood ratio = 5.2 *P* = 0.02
Bipolar disorder type I	3.0 ± 2.7 (*n* = 139)		*n* = 62	*n* = 77	

Results of independent samples T-testing are reported in column 3. We identify significantly greater concentration of CMV IgG in BD-I volunteers as compared to healthy control volunteers (T_234_ = 3.1, *P*_uncorr_ = 0.002, and *P*_corr_ = 0.006). Chi-squared testing confirmed that CMV IgG seropositivity status was associated with 5.2 times greater likelihood of the presence of a diagnosis of BD-I (*P* = 0.02). Neither HSV-1 nor HSV-2 differed significantly between the BD-I and healthy control groups.
